# Translation and adaptation of the person-centered maternity care scale to a Persian-speaking population: a confirmatory factor analysis

**DOI:** 10.1186/s12889-024-19117-1

**Published:** 2024-06-18

**Authors:** Mansoureh Jamshidimanesh, Nafiseh Mohammadkhani

**Affiliations:** Department of Midwifery, School of Nursing and Midwifery, Iran University Medical and Sciences, Tehran, Iran

**Keywords:** Person-centered maternity care, Maternity care, Confirmatory factor analysis, Persian adaptation

## Abstract

**Background:**

Recognized as the most exhaustive multidimensional evaluation of women's person-centered experiences during childbirth, the Person-Centered Maternity Care (PCMC) Scale offers domain-specific insights into facets of care. This instrument has yet to be translated into Persian. Hence, this study purposed to translate and ascertain the reliability and validity of a Persian version of the PCMC scale for postpartum women in Iran.

**Methods:**

A cross-sectional study was facilitated at multiple comprehensive health centers within Tehran, Iran, from February 2022 until July 2022. Postpartum women within seven days after childbirth who were referred to selected comprehensive health centers for newborn thyroid screening were conveniently sampled. The validation process for the questionnaire utilized confirmatory factor analysis (CFA), while it gauged convergent validity via factor loads, average variance extracted (AVE), along with composite reliability (CR). Discriminant credibility was evaluated utilizing HTMT alongside the Fornell-Larcker Criteria. Data analysis procedures were conducted through IBM SPSS Statistics for Windows Version 16 and SMART PLS Statistics for Windows Version 4.0.9.9.

**Results:**

All the items were within the acceptable range of factor loading, except for questions 3 of the facility and 6 of dignity, which were removed from the model. The AVE values for all the variables were above 0.50, and the CR values were above 0.78, indicating convergent validity. On the horizontal loading table, all of the indicators met the conditions. Additionally, the findings validate that the HTMT indicator associated with all constructs remained below 0.9, which confirms divergent relevance about the survey tool under consideration. The composite reliability values also indicated good overall reliability for all the constructs, ranging from 0.78 to 0.91.

**Conclusions:**

The results of the present study indicate that the Persian version of the PCMC is a reliable and valid tool for measuring person-centered maternity care in Persian-speaking populations.

## Background

The process of childbirth represents a seminal transformation in a woman's life, marked not only by physical change but also substantial emotional upheaval. This occurrence is characterized by profound psychological, social, and emotional dimensions that permanently occupy the maternal consciousness [1]. Consequently, adverse experiences during childbirth can yield enduring mental repercussions [2]. During parturition, mothers perceive an inherent vulnerability concerning their well-being and that of their child due to the unpredictability inherent in such events. This perceived vulnerability tends to engender a profound yearning for secure, accommodating, respectful, and reactive care throughout labor and birth [3]. It follows then that maternity care must transcend beyond merely averting morbidity or mortality. Rather it ought to honor fundamental human rights of women—explicitly respecting their autonomy, dignity, sentiments, independent decisions and personal preferences [4].

Regrettably, numerous women encounter healthcare services that do not align with ideal standards [5]. Evolving research findings coupled with experiential and singular case reports gathered from maternity care systems across the globe—ranging from highly affluent societies to economically disadvantaged nations- portray a contrasting and alarming scenario [5]. Substantial efforts on a global level have been targeted at augmenting the quality of services within facilities providing maternal and reproductive health care [6]. Consequently, there have been appeals for an increased emphasis on person-centric reproductive health care [7]. This type of care is considerate towards and adaptable to the preferences, requirements, and values upheld by women and their families [8], aspects which are underscored in The World Health Organization's recommendations aiming for favorable childbirth experiences [9].

In Iran's context specifically, a significant step was taken in formulating the ‘Mothers Bill of Rights’ back in 2003 as an endeavor towards advocating rights bestowed upon mothers during labor [10]. Respectful treatment towards expectant mothers has also been included within Iran’s National Guidelines of Vaginal Childbirth [11]. However, comprehensive studies probing into Iranian women’s experiences reveal that optimality levels regarding to childbirth-care quality are yet to be achieved [12]. For example, some women did not receive timely prevention or detection of complications at birth, did not have access to basic birth facilities, or did not receive enough support, continuity of care, safety, or respect [11, 13]. Based on the evidence, three-quarters of Iranian female respondents reported experiencing disrespectful maternity-related treatments [12]. The potential explanation for the existing levels of respectful maternity care may derive from the non-woman-centric approach to childbirth in Iran, which is primarily focused on medical interventions [14].

The momentum driving efforts towards enhancing and measuring Person-Centered Maternity Care (PCMC) might be inhibited without validated and universally accepted instruments, due to a lack of coherency regarding PCMC's definition and targeted intervention strategies. An objective analysis identified that out of 36 tools that measure women’s birth experiences, only seven had psychometric properties suggestive of high-quality scales. Alarmingly, none were proven effective in low-to-medium-income countries (LMICs) [15].

However, publications made two noteworthy scales to assess women’s birthing experiences in LMICs demonstrating robust validity and reliability: Sheferaw et al.'s 15-item RMC perception scale and Afulani et al.'s comprehensive 30-item PCMC scale [16, 17]. The latter encompasses broader facets of a woman's experience than its counterpart [18]. The rigorous development involving expert reviews, literature examination, and cognitive interviews underscores the substantial content validity rendered by PCMC [19].

To date, the distinguished Person-Centered Maternity Care Scale has been solely validated tool representing as cornerstone accommodating each dimension prescribed by WHO Quality Care Framework equivalent for process indicators predicated onto standardized methodologies inclusive cognitive interviewing alongside psychometric confirmation [19]. The 30-item PCMC, originally developed in Kenya as reported by Afulani and colleagues [17], the scale was later validated in India [20] and consequently tested in Ghana [18]. The tool offers unique insights into respectful maternity care domains and consisted of dignity, autonomy, privacy/confidentiality, communication, social support, trust, supportive care, and the health facility environment subscales [21]. Currently, a Persian adaptation of this questionnaire is nonexistent. Consequently, the objective of our study revolved around translating the PCMC scale to Persian followed by an assessment of its reliability and validity for postpartum women in Iran.

## Methods

### Study design

This methodological study aimed to translate and evaluate the psychometric properties of the PCMC questionnaire. This investigation was divided into two phases. In phase 1, the World Health Organization’s (WHO) process of translation and adaptation of instruments was followed [22]. The process included forward and backward translations, pre-testing including expert panels, and cognitive interviewing with possible participants. In phase 2, the reliability and validity of the Persian version of the PCMC were assessed through a cross-sectional survey.

#### Step 1_Preperation and permission

Permission to use and translate the questionnaire from the Questionnaire Developer (Patience Afulani) was obtained by Email.

#### Step 2_ Forward translation from source to target language

The primary translation of the Person-centered Maternity Care Scale (PCMC) was proficiently executed by a team of three adept bilingual professionals, comprising a midwife, a physician specializing in reproductive health, and an expert in English-to-Persian translation studies. The translators exhibited mastery over both English (source language) and Persian (target language). After undertaking individual translations into written form, the panel integrated their respective versions into one cohesive translation. The culmination of this process resulted in the creation of the preliminary translated scale: Version 1.0 Persian-PCMC.

#### Step 3_Backward translation from target to source language

To ensure accuracy and maintain the original intent of expressions used within PCMC, the following step involved backward translating Version 1.0 into English under another review team consisting primarily of two members—an academic practitioner with expertise in midwifery science and an experienced English instructor—were intentionally kept unaware about specific phrases or terminologies employed within the source material (PCMC for reference). Post translating their assessment into what was referred as "Version 2.0 Backward Translation Persian-PCMC", it underwent comparison analysis to original PCMC context. Any discrepancies between draft copies were addressed at this phase, and finally all back translations were critically reviewed against wordings contained within original English version for practical cross validation.

#### Step 4_Committee review

In a rigorous effort to ascertain the face validity of the provided scale, we incorporated an esteemed group of translators, back-translators, and experts within the subject field to ensure clear interpretation and satisfactory translation conscientiously. The process was further augmented with an in-depth examination of cultural compatibility for Version 1.0 Persian-PCMC. Every dispute encountered throughout this phase was effectively addressed to finalize the translation.

To methodically evaluate both face and content validity of our questionnaire, professional consultations were initiated employing systematic empirical approaches thereby determining indices for Content Validity (CVI) along with Content Validity Ratio (CVR). These calculative methods also included semi-structured cognitive evaluations. CVI is a popularly recognized strategy for measuring content validity during instrument creation. I-CVI is calculated based on multiplying very relevant ratings given by experts per item by the total number of evaluators. Conversely, CVR acts as a measure for deeming item significance or essentiality via associates mathematical formula namely CVR = (Ne – N/2)/(N/2), wherein Ne stands out as total neutral expert evaluation whereas N representing number tallying all panel members.

#### Step 5: Field testing

Following preliminary steps, this section depicted a demonstration draft application over a selective sample size constituting ten patients adhering aptly to research requisite criteria parameters assertion pool who subsequently made necessary interpretations regarding elements such as—comprehensive clarity build-up; ease pertaining straightforward language comprehension; intelligibility indicator in relevance with terminological utilization; simplicity representation alongside achievable efficacies concerning operational survey completion aspects signifying overall response encapsulation. The ten participants consistently expressed that the PCMC scale was straightforward, coherent, and pertinent when evaluating person-centered care in maternity within the Persian context.

### Sample size consideration

Determining an appropriate sample size for structural equation modeling involves using various methodologies and software tools. One such potent tool is Soper software [23], utilized in this study. With an anticipated effect size of 0.19 and a desired statistical power set at 0.9, the number of latent variables included in the model is seven. There are 22 manifest variables corresponding to questions related to these variables; all set at a significance level of 0.01. Taking into consideration initial value stated by the Soper calculator puts us at a base requirement of approximately no less than 272 participants for deciding the impact; thereby necessitating minimum required sample volume be fixed at around 107 people to make up our model appropriately and making our recommended volume come out as equaling or exceeding total count of exactly about 272 individuals. We further considered possibility upon sampling dropout occurrences together with potential missing data situation arising during research tenure process; hence resulting towards setting upper limit cap off mark pegged closely approximating 300 being established as final decided fully computed target working on this specific academic pursuit investigation.

### Instruments

To collect the data, PCMC scale (final version), demographic and obstetrical information forms were used:

### Demographic characteristics and obstetrical information

This form consisted of two parts. Part one included 11 items of socio-demographic information: age, participant and husband occupation and education level, household's income, and residential status. Part two: 11 items of obstetrical information: number of pregnancies and parturitions, infant age, type of hospital, prenatal class attendance, spacing between births, abortion history, pain relief, liquid intake in labor, kind of delivery desired, husband’s preferred type of delivery, and type of infant feeding.

### Person-Centered Maternity Care Scale (PCMC)

The Final version of the 22-item PCMC scale (Persian translated) was administered in the survey. To generate sub-scale scores, the items in that sub-scale were summed. The items under each domain are shown in Table 1. For ease of comparison, the calculated summative score can be normalized to 100 by dividing the calculated score by the total possible scores for the scale and sub-scales and then multiplied by 100, such that each score ranges from 0 to 100 [24].

**Table 1:** Scoring guidance for PCMC sub-scales

DIGNITY & RESPECT		QUESTION			QUESTION
Treated with respect		#3		Involvement in care	#8
Friendly		#4		Able to ask questions	#13
Visual privacy		#7		SUPPORTIVE CARE DOMAIN	
Verbal abuse		#5		Talk about feeling	#14
Physical abuse		#6		Labor support	#17
COMMUNICATION & AUTONOMY				Delivery support	#18
Introduce self		#1		Attention when need help	#15
Called by name		#2		Control pain	#16
Consent to procedures		#9		Trust	#19
Delivery position choice		#10		Water	#22
Explain exams/procedures		#11		safe	#21
Explain medicines		#12		Enough staff	#20
SCALE OR SUB-SCALE	# OF ITEMS	POSSIBLE RANGE OF SUMMATIVE SCORES	SUMMATIVE SCORE FOR SAMPLE	RESCALED SCORE FOR SAMPLE	POSSIBLE RANGE OF RESCALED SCORES
Full PCMC	22	66	W	$\left(\frac{W}{66}\right)*100$	0–100
Dignity & respect	5	15	X	$\left(\frac{X}{15}\right)*100$	0–100
Communication & Autonomy	8	24	Y	$\left(\frac{Y}{24}\right)*100$	0–100
Supportive care	9	27	Z	$\left(\frac{Z}{27}\right)*100$	0-1OO

### Data collection

A meticulous cross-sectional investigation transpired at multiple comprehensive health facilities in Tehran, Iran. The duration of this study spanned from February 2022 to July 2022. The targeted participants were postpartum females who sought newborn thyroid screening services at the preselected integrated healthcare centers. Our participant demographic was carefully chosen through convenience sampling and subsequent eligibility assessments: physically sound women birthing a single child without complications via natural delivery methods with the fetus in cephalic presentation between gestational ages of 37–41 weeks during the concluding week. Our recruitment strategy deliberately omitted individuals with previous C-section history, exposure to recent traumatic life events (such as divorce, death of immediate kin or terminal diagnosis for a family member within preceding three months), past experiences with depression or reported psychological disorders, prominent neonatal defects or those experiencing intellectual disabilities or hearing impairments.

All participating candidates willingly provided their written informed consent after receiving exhaustive information about its content and the guaranteed confidentiality it entailed. This procedure took place in secluded spaces tailored for utmost privacy. To accomplish optimal communication and transparency, an extensively trained midwife initially introduced herself to each potential participant before elucidating on her intent behind conducting these interviews; reiterating that all responses would be untraceable back to any individual source, consequently maintaining anonymity throughout the process. The visiting surveyor's attire and identification badge projected them as an external associate rather than a staff affiliate. Ethical clearance for implementing this survey was granted by the Iran University of Medical Science.

### Data analysis

Data analysis was performed using IBM SPSS Statistics for Windows, Version 16 and SMART PLS Statistics for Windows, Version 4.0.9.9 All the statistical tests were two-tailed, and a p value of less than 0.05 was used to indicate statistical significance. The variables are presented as the frequency and percentage for categorical variables and mean and standard deviation (SD) for continuous variables. To investigate the relationship between obstetrical and sociodemographic data and total PCMC, Pearson and chi-square tests were performed.

SmartPLS's capacity to execute covariance-based structural equation modeling (CB-SEM) bolsters its support for confirmatory factor analysis (CFA). Consequently, SmartPLS-infused CB-SEMs provide an effective platform for conducting a CFA—a statistical methodology crafted to corroborate the foundational factor structure of identified observed variables [25]. Harnessing the power of CFA allows researchers to substantiate hypotheses regarding potential relations between observed variables and their hidden latent counterparts [26]. Additionally, graphical representations can be built utilizing SmartPLS. This software is a robust alternative to SPSS and Amos by offering significant advantages [26, 27]. To authenticate our questionnaire's validity, we employed confirmatory factor analysis(CFA), focusing on both convergent and divergent factualness. The measure of convergent validity was made possible through the observation of factor loads and average variance extracted(AVE) [28]. For validation purposes under this conceptuality, the factor load needed to be equal or greater than 0.5 coupled with an AVE above this mark alongside composite reliability outstripping that figure. The Fornell–Larcker criterion paired with cross-loading quantification were chief contributors when defining discriminant integrity [29]. Regrettably though the Fornell-Larcker procedure fell short when tasked with analyzing discriminant integrity accurately leading us resorting towards employing an alternate approach—the multitrait-multimethod matrix—to evaluate such verity [28].

The research demonstrated commendable convergent validity, with the Average Variance Extracted (AVE) of constructs surpassing 0.5. Further evidence of this was provided by the composite reliability superseding the AVE—this ascertains that a significant majority of the questionnaire's constructs coincided with said validity. Supporting rigorous evaluations were also undertaken to confirm divergent validity; this involved utilizing both Fornell-Larker and HTMT indices successfully. As per Fornell–Larker index calculation, there was a notable mean extracted variance obtained from correlating each variable to other larger counterparts—such a result underlines robust divergent validity. The HTMT index further reinforced such validation, evidenced by all construct values being less than 0.9—a lower score typically indicating better divergent precision.

## Results

### Socio-demographic characteristics of study participants

Table 2 presents the socio-demographic characteristics of the women in the total sample. According to the table below, the average age of the investigated mothers was 33 years. Among the 300 women surveyed, 46.3% (139 people) had a university education, 226 (75.3%) were homemakers, 35.3% (106 people) of the participants' husbands had a university education, and 153 people (51.0%) were self-employed. More than half of the mothers (61.3%) had sufficient income, and 50.7% had their own residential house.

**Table 2:** Socio-demographic characteristics among participants (*n* = 300)

Variable	Number	percent
**Age**		
15_20	22	3/7
20_25	46	3/15
25_30	77	7/25
30_35	89	3/29
35_40	48	0/16
40_45	18	0/6
**Education**		
Elementary and lower	67	3/22
Intermediate	93	31
University	139	3/46
**Husband’s education**		
Illiterate	73	7/7
Elementary and lower	66	22
Intermediate	105	35
University	106	3/35
**Work status**		
Housekeeper	226	3/75
Employed	74	24.7
**Husband job**		
Unemployed	10	3/3
Employed	99	0/33
Self-employed	153	0/51
Other	38	7/12
**Income**		
Very Low Income	25	3/8
Low income	91	3/30
Sufficient income	184	3/61
**Residential status**		
Tenant	148	3/49
Owner	152	50/7

Table 3 presents the obstetric characteristics of the women in the total sample. Thirty-one percent had given birth in public teaching hospitals, approximately 28% had delivered in private hospitals, and most of the infants of the mothers participating in the study (73%) were breastfed. About 76% of the mothers and 59% of their husbands desired vaginal delivery during pregnancy. According to the above table, during labor and delivery, 33% of mothers did not receive any methods to reduce labor pains. Among the methods used, breathing techniques were the most common. More than half of the mothers (53.3%) had not participated in birth classes, and 79% of the participants had not consumed liquids during labor or delivery. Most of the mothers had experienced one pregnancy and childbirth and had no miscarriages, and 81.7% had a planned pregnancy during delivery and labor. A total of 32.3% of mothers were accompanied by midwives, and 87.3% of mothers had skin-to-skin contact with their babies after delivery.

**Table 3:** Obstetric characteristics among participants (*n* = 300)

Variable	Number	percent
**Gravid(number)**		
1	117	0/39
2	103	3/34
3	52	3/17
4	19	3/6
Five and more	9	0/3
**Spacing between births**		
1_2	23	7/7
2_3	37	3/12
3_5	66	0/22
More than Five	50	7/16
**Abortion history(number)**		
Non	236	7/78
1	56	7/18
2	7	3/2
Three and more	1	3/0
**Type of hospital**		
Public nonteaching hospital	87	0/29
Public teaching hospital	93	0/31
Private hospital	30	0/10
Semi-government hospital	84	0/28
Charity Hospital	6	0/2
**Prenatal class attendance**		
Yes	140	7/46
No	160	3/53
**accompanying midwife presence**		
Yes	97	3/32
No	203	7/67
**Skin to skin**		
Yes	262	3/87
No	38	7/12
**Pain relief**		
Epidural	21	0/7
Parenteral	29	7/9
Entonox	13	3/4
water immersion	23	7/7
breathing techniques	75	0/25
Massage	35	7/11
Other	3	0/1
Non	101	7/33
**liquid intake in labor**		
Yes	237	0/79
No	63	0/21

The results of our study showed that there were significant relationships between the participants and their husbands’ education and career status, hospital type, birth class attendance, fluid consumption, skin-to-skin contact, receiving pain relief, presence of accompanying midwives, household income, number of pregnancies/parturitions and total PCMC. However, there were no significant relationships between delivery interval, number of abortions, number of participants and their husbands’ desired type of delivery, residential status, and total PCMC.

### Convergent validity

**Fig. 1:**
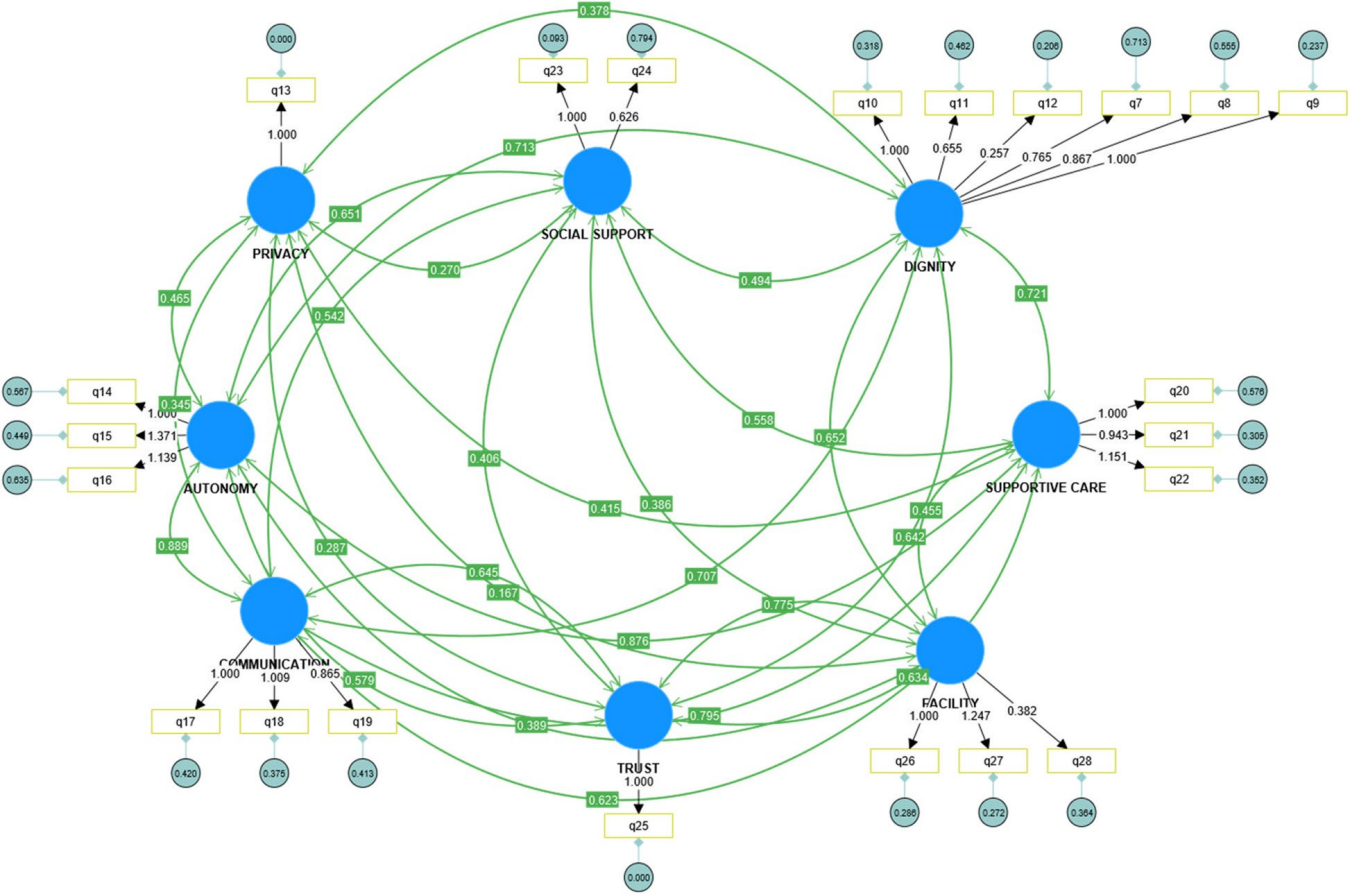
Confirmatory factor analysis of PCMC scale (Primary model)

The measurement scale has convergent validity if the factor loading is greater than or equal to 0.50 [30]. All the items were within the acceptable range except for one item of dignity and one item of facility (Fig. 1).

**Fig. 2:**
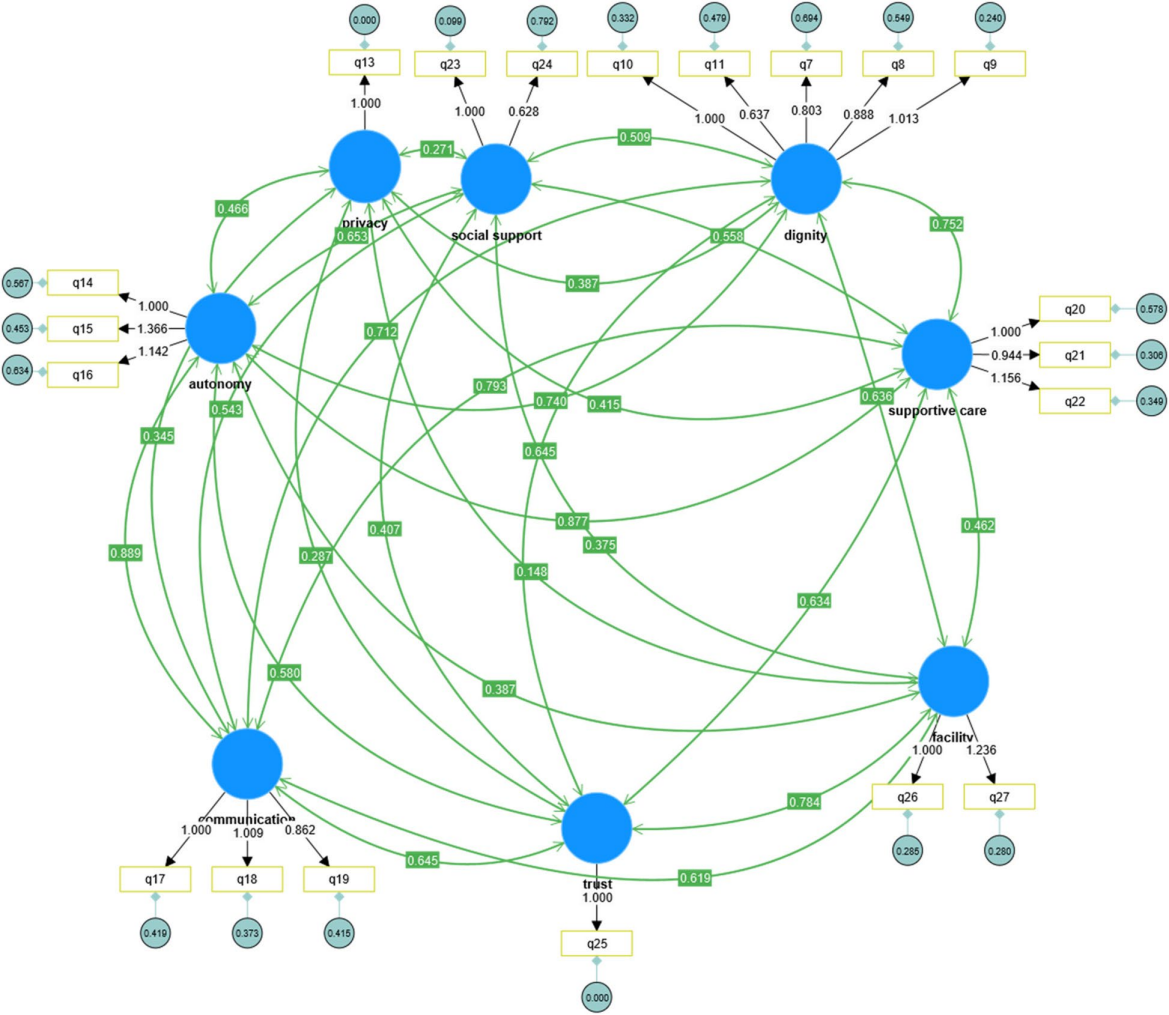
Confirmatory factor analysis of PCMC scale (Final model)

Questions with a factor loading < 0.5 (questions 3 of facility and 6 of dignity) were removed from the model. As a rule of thumb, 20% of the total items can be deleted [31]. The factor loadings of the retained items are shown in Fig. 2.

According to the established criteria, an AVE of 0.50 or above is acceptable. As shown in Table 4, the AVE values for all the variables were above 0.50, and the CR values were above 0.78, indicating convergent validity. The Cronbach’s alpha coefficient of the sub-scale ranged from 0.753 to 0.797.

**Table 4:** Construct reliability and validity of person-centered maternity care scale (*n* = 300)

	**Cronbach's alpha (standardized)**	**Cronbach's alpha (unstandardized)**	**Composite reliability (rho_c)**	**Average variance extracted (AVE)**
**autonomy**	0.787	0.788	0.706	0.501
**communication**	0.795	0.797	0.805	0.578
**dignity**	0.788	0.784	0.791	0.532
**facility**	0.753	0.749	0.758	0.606
**privacy**	1	1	1	1
**social support**	0.789	0.787	0.831	0.697
**supportive care**	0.797	0.793	0.799	0.576
**trust**	1	1	1	1

### Divergent validity

The factor load of each question with its corresponding variable should be at least 0.1 higher than the factor load of that question when it is hypothetically connected to other variables [32]. On the horizontal loading table Table 5, all of the indicators met the conditions, and the indicators of each variable differed from the other variables.

**Table 5:** HTMT ratio

	**Autonomy**	**Communication**	**Dignity**	**Facility**	**Privacy**	**Social Support**	**Supportive Care**	**Trust**
Autonomy								
Communication	0.835							
Dignity	0.785	0.76						
Facility	0.431	0.627	0.607					
Privacy	0.488	0.358	0.413	0.156				
Social Support	0.681	0.564	0.563	0.397	0.245			
Supportive Care	0.806	0.83	0.744	0.444	0.417	0.568		
Trust	0.61	0.658	0.646	0.789	0.287	0.434	0.632	

Additionally, the results in Table ​6 show that the HTMT index for all the constructs was less than 0.9, confirming the divergent validity of the questionnaire [31].

**Table 6:** Fornell & larcker criterion values

Variable	Autonomy	Communication	Dignity	Facility	Privacy	Social Support	Supportive Care	Trust
**Autonomy**	0.661							
**Communication**	0.889	0.76						
**Dignity**	0.74	0.712	0.666					
**Facility**	0.387	0.619	0.636	0.778				
**Privacy**	0.466	0.345	0.387	0.148	1			
**Social Support**	0.653	0.543	0.509	0.375	0.271	0.835		
**Supportive Care**	0.877	0.793	0.752	0.462	0.415	0.558	0.759	
**Trust**	0.58	0.645	0.645	0.784	0.287	0.407	0.634	1

## Discussion

The results of the present study indicate that the Persian version of the PCMC is a reliable and valid tool for measuring person-centered maternity care in Persian-speaking populations. In this study, the translation of the questionnaire was conducted with great care by individuals who were fluent in both the source and target languages and who were knowledgeable about the subject matter. The translation process followed established principles and guidelines, and particular attention was given to ensuring the accuracy and cultural adaptation of the questionnaire. Considering that all hospitals and maternity wards in Iran currently have electricity and water, question 30 of the supportive care subscale, “Was there electricity in the facility?” was removed from the questionnaire, and the item “Was there water in the facility?”, was changed to “Was there warm water in the facility?”. To determine the validity of the form and content, which was done with the Lawshe method and with the opinion of 8 experts, questions 5,18, 23, 26, and 30 of the supportive care subscale, 8 of the dignity and respect subscale, 13 of the communication and autonomy subscale, (How did you feel about the amount of time you waited? Do you feel your health information was or will be kept confidential in this facility? Did the doctors, nurses, or other staff at the facility speak to you in a language you could understand? Did the doctors, nurses, or other staff at the facility support your anxieties and fears? Did you feel the doctors, nurses, or other staff at the facility took the best care of you? Thinking about the labor and postnatal wards, did you feel the health facility was crowded? Was there electricity in the facility) obtained a CVR below 0.75, they were excluded from the questionnaire. To determine the CVI, the calculation of the content validity ratio was used, and questions 5, 8, 13, 18, and 23 obtained a CVI of less than 0.70 and were discarded based on the Lawshe Table [33]. Based on these findings, according to the experts' opinions, eight questions were removed from the above questionnaire, and the total CVI was calculated as 0.87. Items 6 and 22 (Did you feel like you were mistreated? Was there warm water in the facility?) the factor loads were less than 0.5 were excluded from the model. Therefore, the analysis provides support for the use of a 20-item multidimensional PCMC scale in Iran in comparison to the use of a 30-item scale derived from Kenya and a 27-item scale from India. The possible range of scores for the 20-item scale is therefore from 0 to 60 (compared to 0 to 90 for the 30 items and 0 to 81 for the 27 items).

The research demonstrated commendable convergent validity, with the Average Variance Extracted (AVE) of constructs surpassing 0.5. Further evidence of this was provided by the composite reliability superseding the AVE—this ascertains that a significant majority of the questionnaire's constructs coincided with said validity. Supporting rigorous evaluations were also undertaken to confirm divergent validity; this involved utilizing both Fornell-Larker and HTMT indices successfully. As per Fornell–Larker index calculation, there was a notable mean extracted variance obtained from correlating each variable to other larger counterparts—such result underlines robust divergent validity. The HTMT index further reinforced such validation, evidenced by all construct values being less than 0.9—a lower score typically indicating better divergent precision. The internal consistency of the Persian version of the PCMC scale was found to be good for most constructs, with Cronbach's alpha coefficients ranging from 0.78 to 0.91. The composite reliability values also indicated good overall reliability for all the constructs, ranging from 0.78 to 0.91. These findings are consistent with the main study and suggest that the Persian version of the PCMC is a reliable tool for evaluating person-centered maternity care in Persian-speaking populations.

The internal consistency coefficient of the “dignity and respect” sub-dimension was 0.780, while it was 0.813 for “communication and autonomy” and 0.840 for “supportive care”; the total internal consistency coefficient was 0.916 (Table 4). Similar results were reported in studies that evaluated the psychometric properties of PCMC. In the original form of the scale developed by Afulani et al.,Cronbach’s α internal consistency coefficient was 0.63 for the “dignity and respect sub-dimension”, 0.73 for the communication and autonomy sub-dimension and 0.72 for the “supportive care” sub-dimension [17]. Turning attention to diverse versions of forms used in distinctive geographical regions— Cronbach’s α internal consistency coefficients in their Indian incarnation yielded figures at four points: 0.70, 0.67, 0.71, and an impressive final coefficient of 0.85 [20]. In contrast, Kenyan forms registered slightly different measures – they recorded results at sequential points including 0.66, 0.78, 0.75, rounding off at a high point value of 0.088 [17]. Consistency remained key within Ghanaian adaptations—with coefficients reported at regular intervals —including: 0.62,0.72,0.66—peaking finally at an elevated level on scale which rated up to 0.084 [18]. Appraising Turkish samples it emerged the "dignity and respect" sub-dimension measured in just over halfway point mark—observing coefficient consistency rating.613. Further probing revealed “communication and autonomy” scaled higher (0.774) alongside “supportive care,” indexed slightly below(0.743) [34]The overall internal consistency figure rated 0.821—instrumental in maintaining the fulcrum of balance. A final assessment on Chinese versions marked impressive Cronbach's alpha coefficients for the full PCMC and other subscales—all, remarkably, ventured beyond the 0.7 mark [19].

## Conclusion

The research provides substantial evidence underscoring the robust psychometric attributes of the PCMC. It ascertains that this tool constitutes a reliable and valid instrument for assessing person-centered maternity care within the Iranian setting. The Person-Centered Maternity Care Scale now serves as an objective, vigorous measure that can be employed by professionals working with Persian-speaking demographics. Furthermore, its application extends value in deciphering elements of person-centered maternity care necessitating intervention attention; it also facilitates performance appraisal of said interventions. Despite the utilization of a rigorous scientific methodology and robust techniques to adapt and examine the effectiveness of the PCMC in a Persian setting, there exist certain limitations. Primarily, the participants in this study were exclusively recruited from hospitals in Tehran, rendering this sample non-representative of other populations in Iran. Nevertheless, the PCMC framework is probably universally applicable, regardless of geographical location or patient attributes. Secondly, due to the sampling process taking place amidst the COVID-19 pandemic, many mothers hurriedly visited health centers, which can potentially influence their responses to the questionnaire.

## Data Availability

The datasets used  during the current study are available from the corresponding author on reasonable request.

## Abbreviations

PCMC: Person-Centered Maternity Care
CFA: Confirmatory Factor Analysis
AVE: Average Variance Extracted
CR: Composite Reliability
HTMT: Heterotrait-Monotrait ratio of correlations
SPSS: Statistical Package for the Social Sciences
PLS: Partial Least Squares
LMIC: Low-to-Medium Income Countries
WHO: World Health Organization
CVI: Content Validity Index
CVR: Content Validity Ratio
SD: Standard Deviation
CFA: Confirmatory Factor Analysis
CB-SEM: Covariance-based Structural Equation Modeling
AmoS: Analysis of a Moment Structures

## References

[1] McKelvin G, Thomson G, Downe S. The childbirth experience: a systematic review of predictors and outcomes. Women and Birth. 2021;34(5):407–16. 33039281 10.1016/j.wombi.2020.09.021

[2] Kranenburg L, Lambregtse-van den Berg M, Stramrood C. Traumatic Childbirth Experience and Childbirth-Related Post-Traumatic Stress Disorder (PTSD): A Contemporary Overview. Int J Environ Res Public Health. 2023;20(4):2775. 36833472 10.3390/ijerph20042775 PMC9957091

[3] Ahmed HM. Role of verbal and non-verbal communication of health care providers in general satisfaction with birth care: a cross-sectional study in government health settings of Erbil City. Iraq Reprod Health. 2020;17:1–9. 10.1186/s12978-020-0894-3 PMC7063718 32151284

[4] Maldie M, Egata G, Chanie MG, Muche A, Dewau R, Worku N, et al. Magnitude and associated factors of disrespect and abusive care among laboring mothers at public health facilities in Borena District, South Wollo, Ethiopia. PLoS ONE. 2021;16(11):e0256951. 34793460 10.1371/journal.pone.0256951 PMC8601571

[5] Stanton ME, Gogoi A. Dignity and respect in maternity care. BMJ Glob Health. 2022;5:2(Suppl 2):e009023. 10.1136/bmjgh-2022-009023. 10.1136/bmjgh-2022-009023 PMC8948344 35318193

[6] Tikkanen R, Gunja MZ, FitzGerald M, Zephyrin L. Maternal mortality and maternity care in the United States compared to 10 other developed countries. Commonw Fund. 2020;10:6.

[7] Attanasio LB, Ranchoff BL, Paterno MT, Kjerulff KH. Person-centered maternity care and health outcomes at 1 and 6 months postpartum. J Women’s Heal. 2022;31(10):1411–21. 10.1089/jwh.2021.0643 PMC9618378 36067084

[8] Rishard M, Fahmy FF, Senanayake H, Ranaweera AKP, Armocida B, Mariani I, et al. Correlation among experience of person-centered maternity care, provision of care and women’s satisfaction: cross sectional study in Colombo, Sri Lanka. PLoS ONE. 2021;16(4):e0249265. 33831036 10.1371/journal.pone.0249265 PMC8031099

[9] de Masi S, Bucagu M, Tunçalp Ö, Peña-Rosas JP, Lawrie T, Oladapo OT, et al. Integrated person-centered health care for all women during pregnancy: implementing World Health Organization recommendations on antenatal care for a positive pregnancy experience. Glob Heal Sci Pract. 2017;5(2):197–201. 10.9745/GHSP-D-17-00141 PMC5487083 28655799

[10] Mirlohi V, Ehsanpour S, Kohan S. Health providers’ compliance with pregnant women’s Bill of Rights in labor and delivery in Iran. Iran J Nurs Midwifery Res. 2015;20(5):565. 26457093 10.4103/1735-9066.164503 PMC4598902

[11] Moridi M, Pazandeh F, Potrata B. Midwives’ knowledge and practice of Respectful Maternity Care: a survey from Iran. BMC Pregnancy Childbirth. 2022;22(1):1–8. 36199103 10.1186/s12884-022-05065-4 PMC9535863

[12] Hajizadeh K, Vaezi M, Meedya S, Mohammad Alizadeh Charandabi S, Mirghafourvand M. Iranian mother’s perspectives about aspects and determinants of disrespect and abuse during labor and delivery: a qualitative study. Women Health. 2023;63(8):623–36. 37643996 10.1080/03630242.2023.2250466

[13] Pazandeh F, Potrata B, Huss R, Hirst J, House A. Women’s experiences of routine care during labour and childbirth and the influence of medicalisation: a qualitative study from Iran. Midwifery. 2017;53:63–70. 28763721 10.1016/j.midw.2017.07.001

[14] Haghdoost S, Abdi F, Amirian A. Iranian midwives’ awareness and performance of respectful maternity care during labor and childbirth. Eur J midwifery. 2021;5:59. 35083427 10.18332/ejm/143873 PMC8711250

[15] Afulani PA, Buback L, McNally B, Mbuyita S, Mwanyika-Sando M, Peca E. A rapid review of available evidence to inform indicators for routine monitoring and evaluation of respectful maternity care. Glob Heal Sci Pract. 2020;8(1):125–35. 10.9745/GHSP-D-19-00323 PMC7108935 32234844

[16] Sheferaw ED, Mengesha TZ, Wase SB. Development of a tool to measure women’s perception of respectful maternity care in public health facilities. BMC Pregnancy Childbirth. 2016;16:1–8. 27026164 10.1186/s12884-016-0848-5 PMC4810502

[17] Afulani PA, Diamond-Smith N, Golub G, Sudhinaraset M. Development of a tool to measure person-centered maternity care in developing settings: validation in a rural and urban Kenyan population. Reprod Health. 2017;14(1):1–18. 28938885 10.1186/s12978-017-0381-7 PMC5610540

[18] Afulani PA, Phillips B, Aborigo RA, Moyer CA. Person-centred maternity care in low-income and middle-income countries: analysis of data from Kenya, Ghana, and India. Lancet Glob Heal. 2019;7(1):e96–109. 10.1016/S2214-109X(18)30403-0 PMC6293963 30554766

[19] Naito YT, Fukuzawa R, Ganchimeg T, Afulani PA, Aiga H, Kim R, et al. Validation of the person-centered maternity care scale at governmental health facilities in Cambodia. PLoS ONE. 2023;18(7):e0288051. 37410783 10.1371/journal.pone.0288051 PMC10325110

[20] Afulani PA, Diamond-Smith N, Phillips B, Singhal S, Sudhinaraset M. Validation of the person-centered maternity care scale in India. Reprod Health. 2018;15:1–14. 30157877 10.1186/s12978-018-0591-7 PMC6114501

[21] Zhong X, Hu R, Afulani PA, Li X, Guo X, He T, et al. Cross-cultural adaptation and psychometric properties of the Chinese version of the Person-Centered Maternity Care Scale. BMC Pregnancy Childbirth. 2023;23(1):652. 37689683 10.1186/s12884-023-05959-x PMC10492356

[22] World Health Organization. World Health Organization Process of Translation and Adaptation of Instruments. [Internet]. 2020. Available from: http://www.who.int/substance_abuse/research_tools/translation/en/

[23] Ramos-Vera CA. Beyond sample size estimation in clinical univariate analysis. An online calculation for structural equation modeling and network analysis on latent and observable variables. Nutr Hosp. 2021;38(6):1310. 34538059 10.20960/nh.03751

[24] University of California, San Francisco I for GHS, University of California, Los Angeles J, KFS of PH. Person-Centered Maternity Care (PCMC) scale guide. 2022. p. 1–8.

[25] Jhantasana C. Using Latent Variables for Confirmatory Composite Analysis. 2022.

[26] Hair JF, Risher JJ, Sarstedt M, Ringle CM. When to use and how to report the results of PLS-SEM. Eur Bus Rev. 2019;31(1):2–24.

[27] Kline RB. Principles and practice of structural equation modeling. New York: Guilford publications. 2023;360.

[28] Cheung GW, Cooper-Thomas HD, Lau RS, Wang LC. Reporting reliability, convergent and discriminant validity with structural equation modeling: a review and best-practice recommendations. Asia Pacific J Manag. 2023;1–39.

[29] Rönkkö M, Cho E. An Updated Guideline for Assessing Discriminant Validity. Organ Res Methods [Internet]. 2020 Nov 23;25(1):6–14. Available from: 10.1177/1094428120968614

[30] Pillai NV. Reliability, validity and uni-dimensionality: a primer. 2020.

[31] Hair Jr JF, Hult GTM, Ringle CM, Sarstedt M, Danks NP, Ray S, et al. Evaluation of reflective measurement models. Partial Least Squares Struct Equ Model Using R A Workb. 2021;1:75–90.

[32] Franke G, Sarstedt M. Heuristics versus statistics in discriminant validity testing: a comparison of four procedures. Internet Res. 2019;29(3):430–47.

[33] Almanasreh E, Moles R, Chen TF. Evaluation of methods used for estimating content validity. Res Soc Adm Pharm. 2019;15(2):214–21. 10.1016/j.sapharm.2018.03.066 29606610

[34] Özşahin Z, Altiparmak S, Aksoy Derya Y, Kayhan Tetik B, Inceoğlu F. Turkish validity and reliability study for the person-centered maternity care scale. J Obstet Gynaecol Res. 2021;47(9):3211–22. 34278667 10.1111/jog.14913

