# Correction: Translation and adaptation of the person-centered maternity care scale to a Persian-speaking population: a confirmatory factor analysis

**DOI:** 10.1186/s12889-024-20175-8

**Published:** 2024-10-08

**Authors:** Mansoureh Jamshidimanesh, Nafiseh Mohammadkhani

**Affiliations:** Department of Midwifery, School of Nursing and Midwifery, Iran University Medical and Sciences, Tehran, Iran

**Correction: BMC Public Health (2024) 24:1619**

10.1186/s12889-024-19117-1

The original publication of this article was missing the acknowledgement of Mansoureh Jamshidimanesh.

The contributions of Mansoureh Jamshidimanesh have been listed in this correction article along with the incorrect information, the original article has been updated.

**Incorrect**

**Acknowledgement**

We would like to thank all women who participated in our study.

**Author contribution**

Nafiseh mohammadkhani wrote the main manuscript text and reviewed the manuscript."

**Competing interests**

The authors declare no competing interests.

**Correct**

**Acknowledgement**

We would like to thank all women who participated in our study. We would like also to thank Dr. Mohsen Moradi who taught PLS-SEM us.

**Author contribution**

NM and MJ did design project, NM gathered data. They analyzed and interpreted the data, wrote the main manuscript text and reviewed the manuscript.

**Competing interests**

The present manuscript is extracted from a master thesis funded by the Research Deputy of Iran University of Medical Sciences, Tehran, Iran. Research Deputy had no role in the design of the study and collection, analysis, and interpretation of data and in writing the manuscript.

